# Acute effects of beetroot juice vs. creatine supplementation on maximal strength, autonomic regulation, and muscle oxygenation during incremental resistance exercise

**DOI:** 10.5114/biolsport.2025.151658

**Published:** 2025-06-06

**Authors:** Atef Salem, Achraf Ammar, Mohamed Kerkeni, Mohamed Ali Boujelbane, Ayse Merve Uyar, Leonard Moritz Köbel, Saranya Selvaraj, Reza Zare, Katie M. Heinrich, Haitham Jahrami, Slim Tounsi, Giuseppe Grosso, Piotr Zmijewski, Wolfgang I. Schöllhorn, Khaled Trabelsi, Hamdi Chtourou

**Affiliations:** 1 Department of Training and Movement Science, Institute of Sport Science, Johannes Gutenberg-University Mainz, 55122 Mainz, Germany; 2 High Institute of Sport and Physical Education of Sfax, University of Sfax, Sfax 3000, Tunisia; 3 Research Laboratory, Molecular Bases of Human Pathology, LR19ES13, Faculty of Medicine of Sfax, University of Sfax, Sfax 3000, Tunisia; 4 Research Laboratory: Education, Motricity, Sport and Health, EM2S, LR19JS01, High Institute of Sport and Physical Education of Sfax, University of Sfax, Sfax 3000, Tunisia; 5 Department of Chemistry, Faculty of Applied Sciences, University of Sri Jayewardenepura, Gangodawila, Nugegoda, Sri Lanka; 6 SRH Campus Hamburg, SRH University of Applied Sciences Heidelberg, 20095 Hamburg, Germany; 7 Department of Kinesiology, Kansas State University, Manhattan, KS 66506, USA; 8 Department of Research and Evaluation, The Phoenix, Denver, CO 80205, USA; 9 Government Hospitals, Manama, Bahrain; 10 Department of Psychiatry, College of Medicine and Medical Sciences, Arabian Gulf University, Manama, Bahrain; 11 Laboratory of Biopesticides (LBPES), Center of Biotechnology of Sfax, University of Sfax, Sfax, Tunisia; 12 Department of Biomedical and Biotechnological Sciences, University of Catania, Catania, Italy; 13 Jozef Pilsudski University of Physical Education in Warsaw, 00-809 Warsaw, Poland; 14 Department of Movement Sciences and Sports Training, School of Sport Science, The University of Jordan, Amman, Jordan; 15 Research Unit, Physical Activity, Sport, and Health, UR18JS01, National Observatory of Sport, Tunis 1003, Tunisia

**Keywords:** Nitrate, Ergogenic effect, Dietary Supplements, EIMD, Strength, Recovery

## Abstract

This study investigated the acute effects of beetroot juice (BJ) and creatine (CR) supplementation on maximal strength, heart rate variability (HRV), and muscle oxygenation during incremental resistance exercise. Eleven physically active males (age = 21.36 ± 1.8 years) completed a randomized, double-blind, placebocontrolled, crossover protocol. Participants ingested either 0.3 g · kg^−1^ of CR or 15 g of beetroot powder (7.26 mmol · L^−1^ or 450 mg of nitrate) two hours before each session. The three testing sessions included bench press and back squat at 60%, 70%, and 80% of one-repetition maximum (1-RM) until failure. Repetition-tofailure, peak velocity, power, Heart rate, and muscle oxygen saturation (SmO_2_) were recorded during both exercises. HRV indices, lower-limb strength performance, blood lactate, and rating of perceived exertion (RPE) were measured pre- and post-session. As intensity increased, maximum repetitions decreased significantly in all exercises and conditions (p < 0.05). Both BJ and CR improved peak velocity compared to placebo (p < 0.05). BJ led to lower peak heart rates at all intensities during BP and only at 80% of 1-RM during BS and higher SmO_2_ across all intensities (p < 0.05) compared to PLA and CR. From pre- to post-session, lactate and RPE increased (p < 0.05) and lower -limb strength performance and HRV declined (p < 0.05), in all conditions, with no significant differences between BJ and CR. Compared to PLA, BJ showed significantly higher Root mean square of successive differences (RMSSD), Standard deviation of normal-to-normal intervals (SDNN), and high frequency (HF) power at both pre- and post-session (p < 0.05). CR supplementation resulted in significantly higher RMSSD values compared to PLA at both pre- and post-session time points (p < 0.001), while HF was significantly elevated only at post-session (p = 0.018), and SDNN showed no significant differences at either time point. Additionally, BJ revealed significantly higher RMSSD than CR at pre-session (p = 0.041). In conclusion, both BJ and CR significantly enhanced peak velocity during incremental resistance exercises compared to placebo. However, BJ provided additional benefits in muscle oxygenation and autonomic nervous system regulation, particularly during high-intensity efforts.

## INTRODUCTION

Strength training, a cornerstone of physical fitness, involves the systematic application of resistance to induce muscular adaptation, enhancing strength, power, and endurance. This modality not only promotes increases in muscle and bone mass [1] but also improves metabolic health, cardiovascular function, and neuromuscular efficiency [2]. As individuals progress in their training, the principle of progressive overload necessitates incremental increases in resistance to sustain adaptation [3]. However, the physiological stress imposed by resistance exercise often leads to transient impairments in performance, marked by declines in power output, muscle oxygenation, and autonomic recovery [4]. To mitigate these effects, athletes and active populations increasingly turn to ergogenic aids, such as beetroot juice (BJ) and creatine (CR), which target distinct biochemical pathways to enhance performance and accelerate recovery.

BJ, rich in dietary nitrate (${\text{NO}}_{3}^{-}$), has garnered increasing interest for its potential to enhance resistance training performance, though research remains limited compared to endurance sports [5–7]. Upon ingestion, ${\text{NO}}_{3}^{-}$ is reduced to nitrite (${\text{NO}}_{2}^{-}$) by oral microbiota and further converted to nitric oxide (NO) under hypoxic conditions, such as those encountered during high-intensity resistance exercise [8]. NO serves as a potent vasodilator, improving blood flow and oxygen delivery to active muscles while also enhancing mitochondrial efficiency by reducing the oxygen cost of ATP production [9, 10]. These mechanisms are particularly relevant to resistance exercise, where localized hypoxia and metabolite accumulation (e.g., lactate, hydrogen ions) can impair contractile function and accelerate fatigue. Initial studies demonstrated that BJ supplementation enhances muscular endurance, strength, and ATP resynthesis efficiency while elevating plasma nitrate levels, likely via NO-mediated pathways [11, 12]. Proposed mechanisms include preferential vasodilation in type II muscle fibers, which improves blood flow and oxygen delivery during high-intensity efforts [10, 12], as well as accelerates post-exercise recovery by optimizing vascular endothelial function [13], lactate clearance [14], and blood pressure regulation [15].

Recent investigations highlight nuanced effects of BJ timing, dosing, and exercise modality. Acute BJ supplementation (6.4–13 mmol. L^−1^ ${\text{NO}}_{3}^{-}$) has been shown to improve mean velocity, power output, and total repetitions during free-weight bench press, though benefits appear more pronounced in lower-body exercises like back squat (BS) [16, 17]. These discrepancies may stem from differences in muscle mass engagement, fiber-type recruitment, or methodological factors A systematic review highlights small but significant ergogenic effects of BJ on repetition-to-failure and mean power/velocity during resistance exercise, though peak power/velocity metrics remain unaffected, with substantial interstudy variability due to differences in exercise protocols, dosing regimens, and individual ${\text{NO}}_{3}^{-}$ bioavailability responsiveness [16]. Notably, BJ’s benefits may arise from fiber-specific mechanisms, including improved ATP resynthesis in type II fibers via increased oxygen delivery and reduced metabolic cost, as well as enhanced neuromuscular efficiency through elevated motor unit firing rates [10, 12, 18].

CR is one of the most extensively researched and widely used ergogenic aids, particularly for high-intensity, short-duration activities such as weightlifting and sprinting [19], is synthesized endogenously from arginine, glycine, and methionine, but it is also obtained through dietary sources [20]. More than 98% of CR is stored in muscle tissue as creatine phosphate (PCr), a critical substrate for rapid ATP regeneration via the creatine kinase system during intense exercise [21]. Supplementation with CR monohydrate—the most extensively studied form—elevates intramuscular PCr, buffers adenosine diphosphate (ADP) accumulation, and supports repeated bouts of high-intensity exercise [19].

CR supplementation (20–30 g/day) increases intramuscular creatine content by up to 20%, with exercise further enhancing this effect through increased blood flow and creatine transporter (CRT) activation [22]. Studies show that exercise-induced blood flow and CRT activity enhance CR uptake in active muscles [23, 24]. CR supplementation consistently improves strength and power, such as bench press and jump squat performance [25, 26], and enhances muscle strength and high-intensity exercise performance [25]. Combining CR with resistance training significantly boosts muscle strength gains in adults under 50 years [27, 28]. Beyond its effects on power output and strength, CR plays a role in cellular hydration, calcium homeostasis, and oxidative stress mitigation, delaying neuromuscular fatigue and improve recovery [29]. The synergistic relationship between exercise and CR uptake Robinson, Sewell [30], who reported a 68% greater increase in muscle CR content when supplementation was paired with single-leg cycling, emphasizing the role of exercise in enhancing CR retention.

A single dose of CR supplementation can enhance physical performance, particularly in strength activities, by elevating intramuscular CR stores and increasing creatine phosphate availability for high-intensity exercise [25, 31]. This improves strength, power, and performance in activities like weightlifting and sprinting [32, 33]. While multiple doses are often used for full muscle saturation, a single dose, especially when timed around exercise, provides immediate benefits for short-duration, high-intensity efforts [34, 35]. However, methodological limitations—such as variations in exercise protocols and supplementation durations—make definitive conclusions difficult. Current consensus remains divided, as mechanistic data explaining how timing affects CR accrual or performance are sparse. Nonetheless, factors such as baseline CR levels, muscle fiber composition, and aging highlight the need for personalized supplementation strategies to maximize ergogenic benefits.

Despite the research on BJ and CR as individual supplements, there is a lack of direct comparisons between their acute effects on resistance training performance. Given that BJ primarily enhances oxygen delivery and muscle efficiency [10]. while creatine directly supports ATP resynthesis and anaerobic performance [36], understanding their distinct and potentially complementary effects is essential. This study aimed to evaluate the impact of acute BJ vs. CR ingestion on maximal strength performance, heart rate variability (HRV), and muscle oxygenation during an incremental strength testing session. We hypothesized that acute CR supplementation would lead to greater maximal strength performance than BJ or PLA during incremental strength testing. Conversely, we hypothesized that BJ would increase muscle oxygenation and improve HRV parameters during and after exercise, but CR and PLA would produce no significant HRV changes.

## MATERIALS AND METHODS

### Population

A priori power analysis (G*Power v. 3.1.5.1) indicated that 7 participants are required. This calculation was based on a medium effect size (Cohen’s f = 0.5) [37], an alpha level of 0.05, a β-value of 0.8 for repeated measures within factors ANOVA design, and a correlation among repeated measures value of 0.5.

Eleven physically active males (age = 21.36 ± 1.8 years; BMI = 21.29 ± 2.29 kg/m^2^) who were performing structured resistance training (≥ 3 ×/week for ≥ 6 weeks) before the start of the study and were familiarized with BP and BS exercises were recruited from the same gym club. Participants were excluded from the study if they were taking medications that could affect muscle biology (i.e., corticosteroids), had ingested creatine monohydrate or dietary supplements containing creatine, or any type of nutritional substance, supplement, or anabolic substances in the month before or planned to do so during the study. Additionally, Participants were excluded if they were following any specific diet or nutritional regimen (e.g., ketogenic, vegetarian, or intermittent fasting) that could affect the study outcomes. Individuals with pre-existing kidney or liver abnormalities, low blood pressure (< 120/80 mmHg), and any musculoskeletal injuries that could prevent them from following the exercise protocol also were excluded. These inclusion criteria were verified by personal interviews. Participants who did not complete all stages of the experimental protocol or did not adhere to the supplementation protocol described were also excluded.

Participants were asked not to change their habitual diet or consume non-steroidal anti-inflammatory drugs. Additionally, they were asked to avoid both strenuous physical activity and any form of resistance training during the experimental period, as these activities could influence muscle protein turnover [38]. Participants were informed of the risks, potential benefits, and purposes of the study before written consent was obtained. The study was conducted according to the guidelines of the Helsinki Declaration for Human Research. Ethical approval was obtained from the local Research Ethics Committee of the High Institute of Sport and Physical Education of El Kef, El Kef, Tunisia (ISSEPK-0033/2024).

### Experimentation protocol

The study involved an experimental randomized, double-blind, placebo-controlled, crossover trial. We used a within-subject, counterbalanced crossover design to minimize the impact of variation among individuals and order-related effects. The experimental procedure consisted of four visits (1 familiarization session and three test sessions), separated from each other by 7 days, to achieve the total elimination of any effects caused by the beetroot [39] or creatine [40] supplementation, as well as the optimal recovery of the participants [41]. The experimental test measurements were taken in the afternoon hours to achieve optimal strength performance [42, 43]. All testing sessions were conducted at a temperature of 24°C (± 1°C) and at the same time of day (~16:00) to standardize the influence of the circadian rhythm [44, 45].

**FIG. 1:**
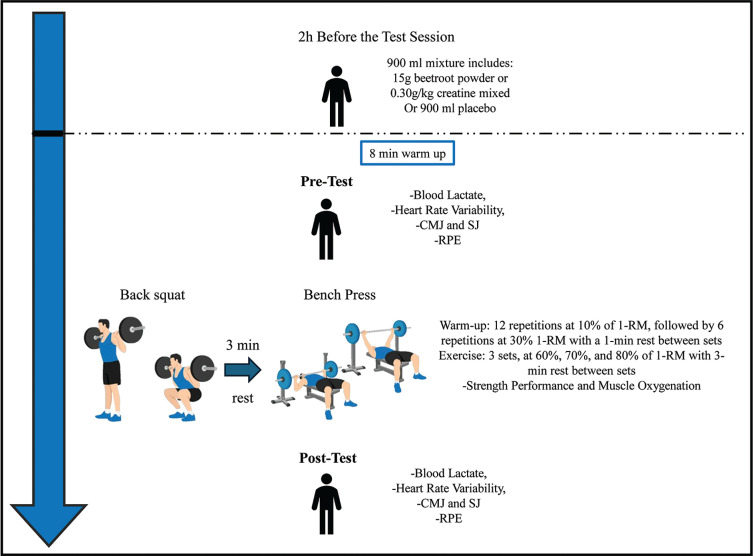
Experimental design. Note: PLA: Placebo; 1-RM: One Repetition Maximum; RPE: Rate of perceived exertion; HRV: Heart Rate Variability; CMJ: Countermovement jump; SJ: Squat jump.

During the first visit, each participant’s anthropometric measures were recorded using bioimpedance (Tanita MC-780MA; Tanita Corporation, Japan). In addition, a technique familiarization of the exercises and materials were performed, and the maximum loads the participant could lift (1-RM) in both the BS [39] and BP exercises were identified. Later, for each supplementation protocol (CR, BJ, and PLA), each participant returned to the laboratory 3 more times to perform an incremental resistance training test to assess the acute ergogenic effect of the supplementations. In total, each participant completed one familiarization and three test sessions over a three-week period (Figure 1), all testing sessions were supervised by the same researcher to minimize variability in instruction, monitoring, and encouragement.

### Familiarization Protocol and One-Repetition Maximum Testing

Participants first participated in a familiarization session for the lifting protocol, focusing on achieving maximum explosive velocity during both the BS and BP. To determine their one-repetition maximum (1-RM), a standardized warm-up was conducted, following the guidelines from previous studies on the effects of BJ on strength performance [16, 37]. The 1-RM testing protocol was conducted according to the ASEP recommendations for accurately assessing muscular strength and power [46]. The BS was performed first, followed by the BP; both exercises were conducted with Smith machine, with a 3-minute rest interval between exercises. During the first set, participants completed five repetitions using 50% of their estimated 1-RM. The second set consisted of three repetitions at 70% of the estimated 1-RM, with 3-minute rest intervals between sets. Following the second set, a 3-minute rest was taken. They then made up to five attempts to reach their 1-RM, with 3-minute rest periods between trials. Since movement velocity was tracked throughout the protocol, the velocity of the 1-RM load was confirmed to match the speed associated with a 1-RM for each exercise [47, 48]. This protocol was then used to establish intensity percentages for the subsequent exercise testing sessions.

### Supplementation protocol

In this study, Creatine monohydrate (GymBeam GmbH, Berlin, Germany) and Bio beetroot powder (Beta vulgaris) (GymBeam GmbH, Berlin, Germany) were consumed in powder form, added to placebo juice (GymBeam GmbH, Berlin, Germany), which was depleted of nitrate and creatine. All supplement drinks (creatine-added, beetrootadded, and placebo) were similar in color, texture, and appearance. The purity of GymBeam beetroot was established by independent laboratory testing (CBS, University of Sfax, Tunisia). Two individuals, not involved in any other aspect of the study, were responsible for randomizing participants and preparing participant study kits. These kits included each participant’s supplement for the duration of the study, detailed supplementation instructions, measuring spoons, and a water bottle. Randomization was conducted with free-online software resource, and the specific sequence for each participant (i.e., the order in which the BJ, CR, or PLA supplements were consumed) was known only by the researchers after all data had been collected.

In accordance with evidence-based research, the CR supplementation dosage was 0.3 g · kg^−1^ · d^−1^ [40]. On the experimentation day, this dose was taken 2 hours prior to the testing session. The BJ supplement contained 7.26 mmol · L^−1^ or 450 mg ${\text{NO}}_{3}^{-}$ per daily dose (corresponding to 15 g of beetroot powder), as tested by the external laboratory. This quantity of ${\text{NO}}_{3}^{-}$ was sufficient to generate an ergogenic effect [49]. Detailed nutritional facts of the used beetroot powder are provided in Table 1. Similar to the CR ingestion protocol, the BJ dose was taken in full 2 hours prior to the testing session [5].

**TABLE 1:** GymBeam BIO Beetroot Powder Nutrition Facts

Nutritional Value	100 g	15 g (Daily dose)
**Values provided by GymBeam**		
**Energy value**	1305 kJ/ 310 kcal	196 kJ/ 46.5 kcal
**Fats**	0.7 g	0.11 g
**Saturated fats**	0 g	0 g
**Carbohydrates**	75 g	11 g
**Sugar**	53 g	8 g
**Protein**	12 g	1.8 g
**Salt**	0.36 g	0.05 g
**Vitamin B1**	5.6 g	0.84 g
**Iron**	37 g	0.55 g
**Mangan**	2.7 g	0.4 g

**Values provided by the Centre of Biotechnology of Sfax**		
**Sodium**	1510 mg	226.5 mg
**Potassium**	2690 mg	403.5 mg
**Magnesium**	210 mg	31.5 mg
**Chloride**	2260 mg	339 mg
**Nitrate**	3000 mg	450 mg
**Phosphate**	1060 mg	159 mg
**Sulfate**	360 mg	54 mg

CR and BJ powder doses of powder were mixed with 900 mL of placebo juice. Accordingly, participants consumed 900 mL of the solution containing 0.3 g · kg^−1^ of CR or 15 g of beetroot powder prior to the testing session. Table 2 provides a detailed description of each supplementation protocol.

**TABLE 2:** Dietary intake 24 hours before the testing session under BJ, CR, and PLA conditions (mean ± SD).

Dietary Intake (Mean ± SD)	PLA	CR	BJ
**Energy (kcal/day)**	2335 ± 285	2370 ± 290	2405 ± 300
**Carbohydrates (g/day)**	299.8 ± 38.6	303.1 ± 39.5	306.5 ± 41.2
**Protein (g/day)**	107.6 ± 16.8	110.2 ± 17.5	111.0 ± 18.0
**Fat (g/day)**	82.1 ± 11.2	84.0 ± 12.0	85.2 ± 12.3

CR-, BJ-, and PLA-based juice were consumed in a glass shaker bottle with gradations (mL) on the side. Participants were instructed to refrain from food or drink (water was permitted ad libitum) for 2 hours before each testing session to ensure a valid estimate of the effects of intra-workout CR supplementation. To avoid any potential confounding influence of usual ${\text{NO}}_{3}^{-}$ intake, participants were provided with a list of foods rich in ${\text{NO}}_{3}^{-}$ (e.g., beetroot, celery, or spinach) to avoid for 48 hours before each testing session.

To maintain dietary consistency, participants completed a 24-hour dietary recall before each test session [16]. These recalls were verbally conducted and recorded by a trained researcher to identify any deviations from dietary instructions, with particular attention to nitrate intake and consumption of substances that could interfere with oral nitrate reduction. All participants were instructed to: (i) eliminate the ingestion of foods rich in ${\text{NO}}_{3}^{-}$, creatine, stimulants (e.g., caffeine), any other type of dietary supplements, gum or sweets, or alcohol that could alter the oral microbiota during the three days before the laboratory visit; (ii) avoid brushing their teeth on the morning of testing and not use antiseptic rinses from one week before the first laboratory visit and throughout the study, due to the potential prevention of the desired increase in nitrite levels after ${\text{NO}}_{3}^{-}$ ingestion [50]; (iii) ensure an adequate level of hydration; (iv) avoid strenuous exercise during the research period; (v) sleep for at least 8 hours per night.

### Strength exercise protocol

During each visit, participants began with an 8-minute standardized supervised warm-up, which involved light muscle activation on a treadmill. After the warm-up, they proceeded with the BS followed by the BP, with a 3-minute rest between the two exercises. Prior to the incremental test, participants completed a standardized warm-up for both exercises, starting with 12 repetitions at 10% of their 1-RM, followed by 6 repetitions at 30% of their 1-RM, with a 1-minute rest between sets [11, 16, 37].

After the warm-up, participants rested for 2 minutes before beginning the incremental strength test. The test consisted of three sets with increasing load intensities at 60%, 70%, and 80% of their 1-RM, where participants performed repetitions until muscle failure. A 3-minute rest period was allowed between sets to ensure recovery from muscle fatigue and maximize the number of repetitions in each set [51]. Both the BS and BP were performed with a full range of motion—knee flexion and extension for the squat and elbow flexion and extension for the BP [52]. Participants were encouraged to execute the concentric phase of each repetition at maximum velocity to ensure optimal muscle strength [48]. The entire testing session, including the warm-up, took approximately 35 minutes.

### Measurements

#### Incremental strength test and performance

During the study, the load in kilograms lifted and the maximum number of repetitions at 60%, 70%, and 80% of 1-RM until failure (for both the BS and BP) were recorded, in addition to the maximum velocity (MV) and maximum power (MP) in watts (W) for each set. The execution velocity and power were monitored by the researchers using a recently validated accelerometer-based sensor, the “Vmax pro.” This sensor demonstrated high validity compared to Vicon (R^2^ = 0.935) and T-Force (R^2^ = 0.968) during deep squat movements [53].

### Muscle oxygenation

To evaluate the oxygenation levels of muscles, we employed Near-Infrared Spectroscopy (NIRS), a non-invasive technique that helps monitor the availability and utilization of oxygen in tissues. With NIRS, we were able to obtain semi-quantitative measurements of oxygen levels in both hemoglobin and myoglobin (tissue O_2_ stores), as well as the volume of hemoglobin present in the muscles [54]. We used a validated NIRS non-invasive device, the Moxy 3-Sensor Bundle (Fortiori Design LLC, USA), which measures real-time tissue oxygen saturation (SmO_2_) [55]. This parameter indicates the percentage of oxygen saturation in the muscle tissue, reflecting the balance between oxygen delivery and utilization in the muscles. Additionally, the total concentration of hemoglobin (THb) in the measured area, including both oxygenated and deoxygenated hemoglobin, was measured, providing information about blood volume and blood flow changes in the muscle.

### Blood lactate Measurement:

Blood lactate levels were measured before, immediately after, and 3 minutes following the testing session [56]. Objective measurements were carried out using the Lactate Pro 2 device Lactate Pro 2 (AKRAY Europe B.V. Prof J.H Bavincklaan 51,183 AT, Amstelveen, the Netherlands). Samples were obtained from the ear lobe, a conventional sampling site [57], after the lobe was cleansed and sterilized with 70% ethanol. The Lactate Pro 2 device used for lactate assessment has a reported coefficient of variation (CV) of approximately 3%, indicating good measurement precision [58].

### Heart Rate Variability (HRV) Monitoring:

HRV was monitored 5 minutes before the testing bout, during the testing bout, and 5 minutes following the testing bout. HRV parameters were measured using a Polar H10 heart rate monitor with a Pro Strap. The data were recorded and analyzed using the Elite HRV app [59]. Several time- and frequency-domain parameters were calculated.

From the time domain, the most used parameters for short-term analysis, including the mean (MeanRR), the standard deviation (SDNN), and the root mean square of successive differences (RMSSD) of the RR intervals, low frequency (LF) and high frequency (HF), and LF:HF ratio were analyzed. Additionally, peak HR during exercises were also recorded. For frequency domain analysis, the lowfrequency (LF, 0.04–0.15 Hz) and high-frequency (HF, 0.15–0.4 Hz) and the LF/HF ratio were computed.

### Ratings of Perceived Exertion (RPE)

RPE values were obtained on a scale of 1–10 immediately at the end of the protocol test [60]. Specifically, participants were instructed to report their perceived exertion immediately after completing the protocol test. They were informed that a numerical value of 2 corresponds to “easy,” 3–4 to “somewhat easy,” 6 to “somewhat hard,” 8–9 to “hard,” and 10 to “extremely hard.”

### Statistical analysis

Descriptive statistics were presented as Mean ± standard deviation (SD). The normality of the data was checked using the Shapiro-Wilk test. To assess the statistical differences between supplementation conditions (BJ vs. CR vs. PLA) and intensity (60% vs. 70% vs. 80%) for strength test performance and between supplementation conditions (BJ vs. CR vs. PLA) and time (pre- vs. post-session) for HRV indices, lower limb strength, lactate, and RPE, a two-way ANOVA (when a parametric test was appropriate) or the LD-F2 model (when a nonparametric test was appropriate) were performed. This model provided an ANOVA-type statistic for condition, time, and the interaction between condition and time. When significant main or interaction effects were found, a post-hoc pairwise comparison with t-test or Wilcoxon test, both with Bonferroni adjustment, were performed. For HRV indices, lower limb strength, lactate, and RPE, the delta change (∆*pre-post*) was calculated as follows: ∆*pre–post* (%) = ((score at post-session − score at pre-session)/score at pre-session) × 100. To assess the difference between delta changes in all variables, the oneway ANOVA (when a parametric test was appropriate) or the Friedman test (when a non-parametric test was appropriate) were performed, followed by a post-hoc pairwise comparison t-test or Wilcoxon test, both with the Bonferroni adjustment. Effect size statistics were calculated as partial eta-squared ($\eta_{\text{p}}^{2}$) for ANOVA and LD.F2 models to assess the magnitude of the effects as small (0.01), moderate (0.06), and large (0.14) [61]. Additionally, Kendall’s coefficient of concordance (w) measures effect size in the Friedman test, estimating agreement among raters as slight (0.20), fair (0.40), moderate (0.60), or almost perfect (0.80) [62]. Standardized effect size (Cohen’s d) analysis was used to interpret the magnitude of differences between means and classified according to Hopkins [63] as : trivial (d ≤ 0.20), small (0.20 < d ≤ 0.60), moderate (0.60 < d ≤ 1.20), large (1.20 < d ≤ 2.0), very large (2.0 < d ≤ 4.0), and extremely large (d > 4.0).

Significance was accepted for all analyses at the a priori level of p < 0.05. Statistical analyses were conducted using the R programming language [64]. ANOVA models for normally distributed data were conducted with the “afex” package [65]. The LD-F2 model was performed with the “nparLD” package [66]. Friedman tests and pairwise comparisons with t-tests and Wilcoxon tests were conducted with the “rstatix” package [67]. The visualization was conducted with the “ggplot2” package [68].

## RESULTS

### Dietary intake

Dietary intake for both BJ and PLA conditions were presented in Table 2. No significant differences in energy or macronutrient intake were found between conditions (p > 0.05).

### Incremental strength test

#### Repetition-to-failure

The ANOVA model analyses of the maximum repetitions achieved in the BP and BS revealed significant main effects for supplementation condition and intensity, but no interaction was found (Table 3).

**TABLE 3:** ANOVA/LD.F2 Results for Strength Test Performances in Bench Press and Back Squat at Incremental Intensities, with Differences by Supplementation and Comparisons Between Conditions and Intensities.

	Supplementation Condition	BP			ANOVA/LD.F2	BS			ANOVA/LD.F2
	
		60%	70%	80%		60%	70%	80%	
**Maximum Repetitions**	**PLA**	12.73 ± 2.94	7.27 ± 1.9 *	3.09 ± 1.45 *¤	**SC:** F_(1.77, 17.70)_ = 7.04, p = 0.007, $\eta_{\text{p}}^{2}$ = 0.413	41.36 ± 20.98	29.82 ± 15.06	17.91 ± 10.98 *	**SC:** F_(1.42, 14.20)_ = 5.77, p = 0.022, $\eta_{\text{p}}^{2}$ = 0.37

	**CR**	15.09 ± 4.13	10.18 ± 4.35 *	4.18 ± 2.18 *¤	**I:** F_(1.85, 18.55)_ = 198.29, p < 0.001, $\eta_{\text{p}}^{2}$ = 0.952	60.73 ± 26.35	34.27 ± 13.87 *	24.27 ± 13.12 *	**I:** F_(1.55, 15.46)_ = 48.80, p < 0.001, $\eta_{\text{p}}^{2}$ = 0.83

	**BJ**	15.45 ± 4.41	9.18 ± 3.74 *	5 ± 3.03 *¤	**SC × I:** F_(2.87, 28.66)_ = 0.97, p = 0.418, $\eta_{\text{p}}^{2}$ = 0.088	58.64 ± 27.61	39 ± 22.38	27 ± 13.65 *	**SC × I:** F_(2.68, 26.84)_ = 2.19, p = 0.118, $\eta_{\text{p}}^{2}$ = 0.18

**Peak Velocity (m · s^-1^)**	**PLA**	0.56 ± 0.08	0.44 ± 0.07 *	0.32 ± 0.06 *¤	**SC:** F_(1.64, ∞)_ = 40.75, p < 0.001, $\eta_{\text{p}}^{2}$ = 0.80	0.55 ± 0.1	0.49 ± 0.06	0.35 ± 0.06 *¤	**SC:** F_(1.59, ∞)_ = 37.18, p < 0.001, $\eta_{\text{p}}^{2}$ = 0.79

	**CR**	0.66 ± 0.07	0.54 ± 0.07 *	0.48 ± 0.06 *^a^	**I:** F_(1.87, ∞)_ = 72.48, p < 0.001, $\eta_{\text{p}}^{2}$ = 0.88	0.72 ± 0.09 a	0.58 ± 0.07 *	0.5 ± 0.06 *¤^a^	**I:** F_(1.59, ∞)_ = 119.26, p < 0.001, $\eta_{\text{p}}^{2}$ = 0.92

	**BJ**	0.72 ± 0.07 a	0.56 ± 0.08 *^a^	0.5 ± 0.08 *^a^	**SC × I:** F_(3.09, ∞)_ = 0.10, p = 0.961, $\eta_{\text{p}}^{2}$ = 0.01	0.64 ± 0.11	0.59 ± 0.09 a	0.46 ± 0.09 *¤^a^	**SC × I:** F_(2.7, ∞)_ = 1.94, p = 0.127, $\eta_{\text{p}}^{2}$ = 0.16

**Peak Power (W)**	**PLA**	508.24 ± 61.07	577.91 ± 57.73 *	767.99 ± 66.19 *¤	**SC:** F_(1.54, 15.38)_ = 0.90, p = 0.401, $\eta_{\text{p}}^{2}$ = 0.08	878.67 ± 49.87	1078.35 ± 60.52 *	1358.47 ± 81.45 *¤	**SC:** F_(1.83, ∞)_ = 1.58, p = 0.208, $\eta_{\text{p}}^{2}$ = 0.14

	**CR**	510.28 ± 57.17	617.88 ± 53.05 *	787.23 ± 62.66 *¤	**I:** F_(1.97, 19.68)_ = 251.52, p < 0.001, $\eta_{\text{p}}^{2}$ = 0.96	926.34 ± 38.17	1095.93 ± 59.45 *	1331.77 ± 97.25 *¤	**I:** F_(1.91, ∞)_ = 536.64, p < 0.001, $\eta_{\text{p}}^{2}$ = 0.98

	**BJ**	503.09 ± 69.57	594.65 ± 58.3 *	787.65 ± 49.57 *¤	**SC × I:** F_(2.90, 28.98)_ = 0.31, p = 0.810, $\eta_{\text{p}}^{2}$ = 0.03	849.11 ± 46.38 b	1092.39 ± 33.52 *	1347.83 ± 69.78 *¤	**SC × I:** F_(3.08, ∞)_ = 2.09, p = 0.097, $\eta_{\text{p}}^{2}$ = 0.17

**Maximum Displacement (cm)**	**PLA**	46.73 ± 2.57	39.82 ± 3.92 *	37.18 ± 1.66 *	**SC:** F_(1.78, ∞)_ = 0.42, p = 0.634, $\eta_{\text{p}}^{2}$ = 0.04	54.36 ± 2.11	49.73 ± 3.82 *	47.55 ± 2.11 *	**SC:** F_(1.90, ∞)_ = 2.22, p = 0.111, $\eta_{\text{p}}^{2}$ = 0.18

	**CR**	45.27 ± 4	41.18 ± 3.63	38.45 ± 1.44 *	**I:** F_(1.26, ∞)_ = 48.97, p < 0.001, $\eta_{\text{p}}^{2}$ = 0.83	54.64 ± 2.62	50.27 ± 3.1 *	47.27 ± 1.56 *¤	**I:** F_(1.63, ∞)_ = 112.31, p < 0.001, $\eta_{\text{p}}^{2}$ = 0.92

	**BJ**	45.73 ± 2.1	40.18 ± 3.57 *	37.73 ± 1.85 *	**SC × I:** F_(2.76, ∞)_ = 1.05, p = 0.364, $\eta_{\text{p}}^{2}$ = 0.10	56.09 ± 2.91	51.64 ± 2.98 *	47.73 ± 1.49 *¤	**SC × I:** F_(2.59, ∞)_ = 0.21, p = 0.866, $\eta_{\text{p}}^{2}$ = 0.02

**BP**: Bench press; **BS**: Back squat; **PLA**: Placebo; **CR**: Creatine; **BJ**: Beetroot juice; **SC:** main effect of supplementation condition; **I:** Main effect of intensity; **SC × I:** interaction between supplementation condition and intensity;

* : compared to 60%;

¤ : compared to 70%;

a : compared to PLA;

b : compared to CR.

Post-hoc pairwise comparison for the BP exercise revealed a significant decline in repetitions for all supplementation conditions from 60% to 70% (BJ: p = 0.002, d = 1.47; CR: p = 0.012, d = 1.47; PLA: p < 0.001, d = 1.47), 60% to 80% (BJ: p < 0.001, d = 3.17; CR: p < 0.001, d = 3.17; PLA: p < 0.001, d = 3.17), and 70% to 80% of 1-RM (BJ: p = 0.043, d = 1.57; CR: p = 0.002, d = 1.57; PLA: p < 0.001, d = 1.57). Regarding the BS exercise, post-hoc pairwise comparisons for BS showed a significant decrease in repetitions from 60% to 80% of 1-RM for BJ (p = 0.006, d = 1.49), CR (p < 0.001, d = 1.7), and PLA (p = 0.006, d = 1.49). Additionally, CR supplementation demonstrated a significant decline in repetitions from 60% to 70% of 1-RM BS (p = 0.007, d = 0.87).

### Peak velocity

The LD.F2 model revealed significant effects of supplementation condition and intensity on peak velocity in the BP and BS exercise, but no significant interactions were found (Table 3). Pairwise comparisons showed significant decreases for the BJ, CR, and PLA conditions from 60% to 70% (BJ: p = 0.006, d = 1.38; CR: p = 0.015, d = 1.38; PLA: p = 0.006, d = 1.38) and 60% to 80% of 1-RM (BJ: p = 0.003, d = 2.08; CR: p = 0.012, d = 1.83; PLA: p = 0.003, d = 2.08) during the BP. Additionally, peak velocity for the PLA condition also declined significantly from 70% to 80% of 1-RM BP (p = 0.017, d = 0.84). Moreover, the BJ condition had a significantly higher peak velocity compared to PLA at 60% (p = 0.015, d = 1.27), 70% (p = 0.011, d = 1.27), and 80% of 1-RM BP (p = 0.006, d = 1.27). Similarly, the CR condition had a significantly higher peak velocity compared to PLA at 80% of 1-RM during BP (p = 0.006, d = 1.08).

During the BS, pairwise comparisons showed that peak velocity significantly decreased for the BJ and PLA conditions from 60% to 80% of 1-RM (BJ: p = 0.012, d = 1.89; PLA: p = 0.012, d = 1.89) and from 70% to 80% (BJ: p = 0.003, d = 1.30; PLA: p = 0.02, d = 1.3). Also, for the CR condition, there was a significant decline between 60% and 70% (p = 0.024, d = 0.84), 60% and 80% (p = 0.012, d = 1.89), and 70% and 80% of 1-RM (p = 0.027, d = 1.30). Moreover, the BJ condition had a significantly higher peak velocity compared to PLA at 70% and 80% of 1 RM (p = 0.029, d = 0.83 for both 70 and 80% of 1-RM). Finally, the CR condition had a higher peak velocity compared to PLA at 60% (p = 0.018, d = 1.15) and 80% of 1-RM (p = 0.011, d = 1.15).

### Peak power

There was only a significant main effect of intensity for peak power during the BP and BS exercises (Table 3). Pairwise comparisons revealed significant increase in peak power during BP for the BJ, CR, and PLA conditions from 60% to 70% of 1-RM (BJ: p = 0.003, d = 1.52; CR: p < 0.001, d = 1.52; PLA: p = 0.038, d = 1.52), from 60% to 80% (BJ: p < 0.001, d = 4.58; CR: p < 0.001, d = 4.58; PLA: p < 0.001, d = 4.58), and from 70% to 80% of 1-RM (BJ: p < 0.001, d = 3.18; CR: ; PLA: p < 0.001, d = 3.18).

Similarly during BS exercise, the peak power significantly increased for all supplementation conditions from 60% to 70% (BJ: p = 0.003, d = 3.86; CR: p = 0.003, d = 3.86; PLA: p = 0.003, d = 3.86), from 60% to 80% (BJ: p = 0.003, d = 6.65; CR: p = 0.003, d = 6.65; PLA: p = 0.003, d = 6.65), and from 70% to 80% of 1-RM (BJ: p = 0.003, d = 3.76; CR: p = 0.003, d = 3.76; PLA: p = 0.003, d = 3.76). And at 60% of 1-RM, the BJ condition had significantly lower peak power compared to CR (p = 0.015, d = 0.11).

### Maximum displacement

The LD.F2 model analyses of maximum displacement during the BP and BS revealed a significant main effect for intensity, while no significant main effects were observed for supplementation conditions or the interaction between condition and intensity (Table 3). During both the BP and BS exercises, the BJ condition had significant decreases in maximum displacement from 60% to 70% (BP: p = 0.02, d = 1.66; BS: p = 0.014, d = 1.50) and from 60% to 80% of 1-RM (BP: p = 0.011, d = 3.36; BS: p = 0.004, d = 3.42). The CR condition had a significant decrease in maximum displacement during the BS from 60% to 70% (p = 0.015, d = 1.50), from 60% to 80% (p = 0.004, d = 3.42), and from 70% to 80% (p = 0.032, d = 1.15), but decreased only from 60% to 80% of 1-RM during the BP (p = 0.011, d = 3.36). For the PLA condition during both exercises, maximum displacement significantly decreased from 60% to 70% (BP: p = 0.011, d = 1.66; BS: p = 0.025, d = 1.50) and from 60% to 80% (BP: p = 0.011, d = 3.36: BS: p = 0.004, d = 3.42). Additionally, the BJ condition had significant decreases in maximum displacement during the BS from 70% to 80% (p = 0.022, d = 1.15).

### Cardiovascular and Metabolic Responses

#### Peak HR

The LD.F2 model analysis of peak HR during the BP revealed significant main effects for both supplementation condition and intensity, while no interaction was found (Table 4). The pairwise comparisons revealed that peak HR significantly increased for all supplementation conditions from 60% to 70% (BJ: p = 0.008, d = 1.47; CR: p = 0.014, d = 1.47; PLA: p = 0.004, d = 1.47), 60% to 80% (BJ: p = 0.001, d = 3.96; CR: p = 0.004, d = 3.96; PLA: p = 0.004, d = 3.96), and 70% to 80% of 1-RM BP (BJ: p = 0.004, d = 1.56; CR: p = 0.004, d = 1.56; PLA: p = 0.005, d = 1.56). Moreover, the BJ condition had significantly lower peak HR compared to CR and PLA at 60% (CR: p = 0.014, d = 0.88; PLA: p = 0.015, d = 0.92), 70% (CR: p = 0.005, d = 0.88; PLA: p = 0.004, d = 0.92), and 80% of 1-RM (CR: p = 0.005, d = 0.88; PLA: p = 0.009, d = 0.92).

**TABLE 4:** ANOVA/LD.F2 Results for Peak HR, muscle oxygen saturation (SmO_2_), and total hemoglobin (tHb) in Bench Press and Back Squat at Incremental Intensities, with Differences by Supplementation and Comparisons Between Conditions and Intensities.

	Supplementation Condition	BP			ANOVA/LD.F2	BS			ANOVA/LD.F2
	
		60%	70%	80%		60%	70%	80%	
**Peak HR (bpm)**	**PLA**	139.27 ± 3.38	155 ± 6.94 *	164.27 ± 2.33 *¤	**SC:** F(1.59, ∞) = 37.18, p < 0.001, ηp2 = 0.79	177.64 ± 5.92	182.82 ± 4.9	185.18 ± 3.57 *	**SC:** F(1.25, ∞) = 4.04, p = 0.060, ηp2 = 0.29

	**CR**	139.45 ± 6.25	153.36 ± 9.58 *	166.09 ± 3.51 *¤	**I:** F(1.59, ∞) = 119.26, p < 0.001, ηp2 = 0.92	173.18 ± 8.4	182.09 ± 4.06 *	188.91 ± 2.47 *¤	**I:** F(1.45, ∞) = 22.21, p < 0.001, ηp2 = 0.69

	**BJ**	128.55 ± 7.09 ab	139.27 ± 6.66 *^a^^b^	156.64 ± 5.41 *¤^a^^b^	**SC × I:** F(2.70, ∞) = 1.94, p = 0.127, ηp2 = 0.16	171.73 ± 9.39	179.55 ± 6.82 *	184.18 ± 3.46 *^a^^b^	**SC × I:** F(2.60, ∞) = 2.08, p = 0.134, ηp2 = 0.17

**SmO_2_ (%)**	**PLA**	78.06 ± 1.18	77.77 ± 1.24	77.18 ± 1.75	**SC:** F(1.99, ∞) = 53.66, p < 0.001, ηp2 = 0.84	78.22 ± 2.15	78.21 ± 1.57	76.84 ± 2.01	**SC:** F(1.98, ∞) = 55.33, p < 0.001, ηp2 = 0.85

	**CR**	79.96 ± 1.47 a	79.88 ± 1.42 a	79.76 ± 1.51 a	**I:** F(1.86, ∞) = 1.74, p = 0.177, ηp2 = 0.15	79.78 ± 1.91	80.45 ± 2.68 a	79.81 ± 1.4 a	**I:** F(1.83, ∞) = 2.45, p = 0.091, ηp2 = 0.20

	**BJ**	81.47 ± 1.67 ab	80.97 ± 1.57 ab	80.19 ± 1.89 a	**SC × I:** F(3.18, ∞) = 0.35, p = 0.804, ηp2 = 0.03	81.71 ± 1.63 a	82.19 ± 1.34 a	81.33 ± 1.21 ab	**SC × I:** F(2.88, ∞) = 0.36, p = 0.773, ηp2 = 0.03

**tHb (g/dl)**	**PLA**	12.62 ± 0.23	12.52 ± 0.17	12.72 ± 0.25	**SC:** F(1.91, 19.05) = 2.84, p = 0.085, ηp2 = 0.22	12.52 ± 0.21	12.41 ± 0.15	12.49 ± 0.21	**SC:** F(1.52, ∞) = 0.88, p = 0.39, ηp2 = 0.08

	**CR**	12.45 ± 0.27	12.61 ± 0.21	12.48 ± 0.2	**I:** F(1.91, 19.08) = 0.58, p = 0.561, ηp2 = 0.06	12.52 ± 0.18	12.43 ± 0.3	12.58 ± 0.26	**I:** F(1.54, ∞) = 1.65, p = 0.199, ηp2 = 0.14

	**BJ**	12.48 ± 0.21	12.48 ± 0.13	12.49 ± 0.2	**SC × I:** F(3.16, 31.58) = 1.67, p = 0.191, ηp2 = 0.14	12.49 ± 0.3	12.48 ± 0.17	12.55 ± 0.21	**SC × I:** F(3.13, ∞) = 0.18, p = 0.918, ηp2 = 0.02

**BP**: Bench press; **BS**: Back squat; **PLA**: Placebo; **CR**: Creatine; **BJ**: Beetroot juice; **SC:** main effect of supplementation condition; **I:** Main effect of intensity; **SC × I:** interaction between supplementation condition and intensity;

* : compared to 60%;

¤ : compared to 70%;

a : compared to PLA;

b : compared to CR.

The LD.F2 model analysis of peak HR during the BS revealed a significant main effect of intensity, but no significant main effect for supplementation condition or the interaction (Table 4). In the pairwise comparisons, HR for the BJ condition increased significantly from 60% to 70% (p = 0.041, d = 1.05) and from 60% to 80% of 1-RM (p = 0.001, d = 1.87). Similarly, for the CR condition, peak HR significantly increased from 60% to 70% (p = 0.002, d = 1.05), from 60% to 80% (p < 0.001, d = 1.87), and from 70% to 80% of 1-RM (p = 0.022, d = 0.99). Moreover, the PLA condition had a significant increase in peak HR from 60% to 80% of 1-RM (p = 0.003, d = 1.87). Furthermore, the BJ condition had a significantly lower peak HR compared to the CR (p = 0.005, d = 0.34) and PLA conditions (p = 0.032, d = 0.07) for 80% of 1-RM.

### Muscle oxygen saturation (SmO_2_)

The LD.F2 model analysis of SmO_2_ recorded during the BP and BS revealed significant main effects for supplementation condition, while the main effect of intensity and the interaction were not significant (Table 4).

Regarding SmO_2_ during the BP, pairwise comparisons for 60% indicated that the BJ condition had significantly higher values compared to the CR (p = 0.01, d = 0.63) and PLA conditions (p < 0.001, d = 2.02), while the CR condition had significantly higher SmO_2_ than PLA (p = 0.042, d = 1.55). At 70%, the BJ condition had significantly greater SmO_2_ compared to the CR (p = 0.032, d = 0.63) and PLA conditions (p = 0.002, d = 2.02), and the CR condition had significantly higher SmO_2_ compared to PLA (p = 0.003, d = 1.55). For 80% of 1-RM, the BJ condition maintained significantly higher SmO_2_ than PLA (p = 0.019, d = 2.02), and the CR condition was significantly superior to PLA (p < 0.001, d = 1.55).

Concerning SmO_2_ during the BS, pairwise comparisons indicated that the BJ condition had significantly higher SmO_2_ compared to PLA at 60% (p = 0.014, d = 2.33) and 70% (p < 0.001, d = 2.33), and 80% of 1-RM (p < 0.001, d = 2.33), and the BJ condition maintained significantly higher SmO_2_ than CR at 80% of 1-RM (p = 0.019, d = 0.99). Additionally, the CR condition had significantly higher SmO_2_ than PLA at 70% (p = 0.032, d = 1.13) and 80% of 1 RM (p = 0.002, d = 1.13).

### Total Hemoglobin (tHb)

During both the BP and BS, no significant main effect for supplementation condition, intensity, or the interaction between supplementation condition and intensity were found for tHb (Table 4).

### HRV responses

**FIG. 2:**
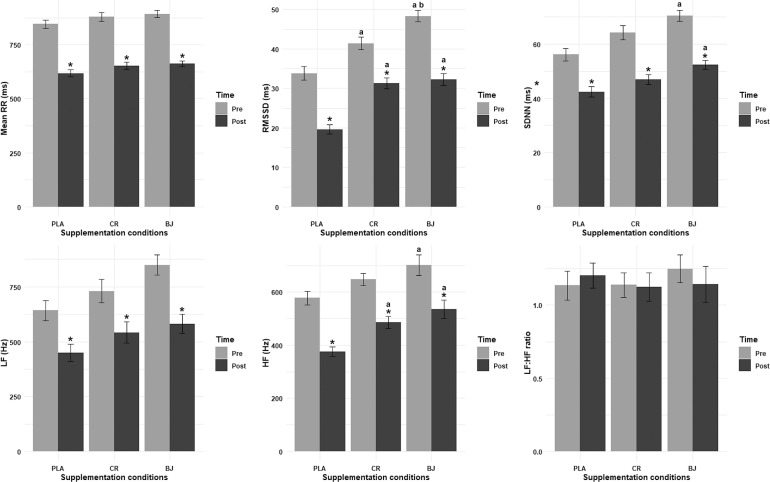
Heartrate variability indices recorded pre- and post-session. PLA: Placebo; CR: Creatine; BJ: Beetroot juice; *: compared to Pre-Session; a: compared to PLA; b: compared to CR.

HRV indices were presented in figure 2.

### Mean RR interval

The LD.F2 model analysis of the mean RR interval revealed significant main effects for supplementation condition (F_(1.98, ∞)_ = 4.72, p = 0.009, $\eta_{\text{p}}^{2}$ = 0.32) and time (F_(1.00, ∞)_ = 181.87, p < 0.001, $\eta_{\text{p}}^{2}$ = 0.95). However, the interaction between supplementation condition and time was not significant (F_(1.76, ∞)_ = 0.14, p = 0.841, $\eta_{\text{p}}^{2}$ = 0.01). Pairwise comparisons demonstrated that the mean RR interval significantly decreased from pre- to post-session in all supplementation conditions (p < 0.001, d = 3.83, d = 3.8, and d = 4.7; respectively for PLA, CR, and BJ).

### RMSSD

The LD F2 model analysis of RMSSD revealed significant main effects for supplementation condition (F_(1.76, ∞)_ = 38.72, p < 0.001, $\eta_{\text{p}}^{2}$ = 0.79) and time (F_(1, ∞)_ = 58.28, p < 0.001, $\eta_{\text{p}}^{2}$ = 0.85), while the interaction between supplementation condition and time was not significant (F_(1.96, ∞)_ = 1.59, p = 0.204, $\eta_{\text{p}}^{2}$ = 0.14). Pairwise comparisons indicated that the BJ condition had significantly higher RMSSD than PLA both pre- (p = 0.006, d = 2.71) and post-session (p = 0.003, d = 2.79), and significantly higher RMSSD than CR pre-session (p = 0.041, d = 1.36). The CR condition also showed a significantly greater RMSSD than PLA both pre- (p = 0.003, d = 1.41) and post-session (p = 0.006, d = 2.79). Moreover, a significant decrease was revealed in RMSSD from pre- to post-session across all supplementation conditions: BJ (p = 0.001, d = 3.19), CR (p = 0.003, d = 2.04), and PLA (p = 0.001, d = 2.92).

### SDNN

The ANOVA model analysis for SDNN revealed a significant main effect for supplementation condition (F_(1.93, 19.35)_ = 14.69, p < 0.001, $\eta_{\text{p}}^{2}$ = 0.6) and time (F_(1, 10)_ = 89.07, p < 0.001, $\eta_{\text{p}}^{2}$ = 0.9), while the interaction between supplementation condition and time was not significant (F_(1.90, 18.99)_ = 0.74, p = 0.486, $\eta_{\text{p}}^{2}$ = 0.07). Pairwise comparisons indicated that the BJ condition had significantly higher values than PLA at both pre- (p < 0.001, d = 1.99) and postsession (p = 0.001, d = 1.71). Moreover, a significant decrease in SDNN was revealed from pre- to post-session across all supplementation conditions; BJ (p < 0.001, d = 3.05), CR (p < 0.001, d = 2.33), and PLA (p < 0.001, d = 1.93).

### LF

The LD.F2 model analysis for LF revealed a significant main effect for supplementation condition (F_(1.73, ∞)_ = 4.25, p = 0.019, $\eta_{\text{p}}^{2}$ = 0.30) and time (F_(1, ∞)_ = 45.33, p < 0.001, $\eta_{\text{p}}^{2}$ = 0.82), while the interaction between supplementation condition and time was not significant (F_(1.89, ∞)_ = 0.57, p = 0.556, $\eta_{\text{p}}^{2}$ = 0.05). Pairwise comparisons revealed a significant decrease in LF values from pre- to post-session for all conditions; BJ (p < 0.001, d = 1.8), CR (p < 0.001, d = 1.13), and PLA (p < 0.001, d = 1.35).

### HF

The ANOVA model analysis of HF revealed significant main effects of supplementation condition (F_(1.62, 16.18)_ = 8.02, p = 0.006, $\eta_{\text{p}}^{2}$ = 0.45) and time (F_(1, 10)_ = 235.28, p < 0.001, $\eta_{\text{p}}^{2}$ = 0.96). However, the interaction between supplementation condition and time was not significant (F_(1.34, 13.44)_ = 0.62, p = 0.492, $\eta_{\text{p}}^{2}$ = 0.06). For the pairwise comparisons for the pre-session condition, the BJ condition demonstrated significantly higher HF values than PLA (p = 0.017, d = 1.04). For the post-session comparisons, the BJ condition had significantly higher HF values compared to PLA (p < 0.001, d = 1.04), and the CR condition also had significantly greater HF values than PLA (p = 0.018, d = 0.77). Moreover, significant reductions were found in HF from pre- to post-session across all supplementation conditions; BJ (p = 0.005, d = 1.36), CR (p < 0.001, d = 2.21), and PLA (p < 0.001, d = 2.79).

### LF/HF ratio

The ANOVA model analysis of the LF/HF ratio revealed non-significant main effects for supplementation condition (F_(1.45, 14.51)_ = 0.14, p = 0.805, $\eta_{\text{p}}^{2}$ = 0.01), time (F_(1, 10)_ = 0.1, p = 0.763, $\eta_{\text{p}}^{2}$ = 0.01), and the interaction between supplementation condition and time (F_(1.89, 18.95)_ = 1.02, p = 0.376, $\eta_{\text{p}}^{2}$ = 0.09).

### Lower limb strength (CMJ and SJ)

**FIG. 3:**
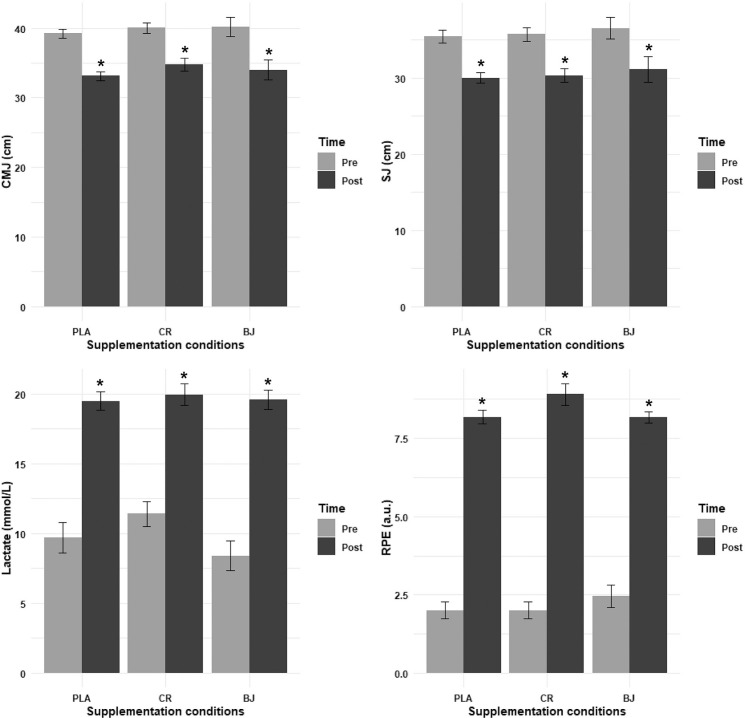
CMJ, SJ, lactate, and RPE recorded pre- and post-session. PLA: Placebo; CR: Creatine; BJ: Beetroot juice; *: compared to Pre-Session.

The ANOVA model analysis of CMJ performance revealed no significant main effect of supplementation condition (F_(1.22, 12.18)_ = 0.45, p = 0.555, $\eta_{\text{p}}^{2}$ = 0.04) and no significant interaction between supplementation condition and time (F_(1.76, 17.61)_ = 1.88, p = 0.184, $\eta_{\text{p}}^{2}$ = 0.16). However, there was a highly significant main effect of time (F_(1, 10)_ = 685.37, p < 0.001, $\eta_{\text{p}}^{2}$ = 0.99), with a significant decrease in CMJ performance from pre- to post-session across all supplementation conditions; BJ (p = 0.006, d = 1.30), CR (p < 0.001, d = 1.97), and PLA (p < 0.001, d = 3.08) (Figure 3).

Regarding the SJ, the ANOVA model analysis revealed no significant main effect of supplementation condition (F_(1.28, 12.78)_ = 0.31, p = 0.644, $\eta_{\text{p}}^{2}$ = 0.03) and no significant interaction between supplementation condition and time (F_(1.42, 14.19)_ = 0.01, p = 0.963, $\eta_{\text{p}}^{2}$ = 0). However, there was a highly significant main effect of time (F_(1, 10)_ = 508.88, p < 0.001, $\eta_{\text{p}}^{2}$ = 0.98), indicating that SJ performance significantly decreased from pre- to post-session across all supplementation conditions; BJ (p = 0.027, d = 1.02), CR (p < 0.001, d = 1.81), and PLA (p < 0.001, d = 2.16) (Figure 3).

### Lactate

The ANOVA model analysis of lactate levels revealed no significant main effect of supplementation condition (F_(1.98, 19.75)_ = 1.42, p = 0.266, $\eta_{\text{p}}^{2}$ = 0.12) and no significant interaction between supplementation condition and time (F_(1.98, 19.8)_ = 1.06, p = 0.366, $\eta_{\text{p}}^{2}$ = 0.10). However, there was a highly significant main effect of time (F_(1, 10)_ = 324.22, p < 0.001, $\eta_{\text{p}}^{2}$ = 0.97). Pairwise comparisons revealed significant increases in lactate from pre- to post-session across all supplementation conditions; BJ (p < 0.001, d = 3.79), CR (p < 0.001, d = 3.06), and PLA (p < 0.001, d = 3.24) (Figure 3).

### Rating of Perceived Exertion (RPE)

The LD.F2 analysis of RPE revealed no significant main effect of supplementation condition (F_(1.94, ∞)_ = 0.60, p = 0.546, $\eta_{\text{p}}^{2}$ = 0.06) and no significant interaction between supplementation condition and time (F_(1.50, ∞)_ = 1.37, p = 0.253, $\eta_{\text{p}}^{2}$ = 0.12). However, there was a highly significant main effect of time (F_(1, ∞)_ = 488.16, p < 0.001, $\eta_{\text{p}}^{2}$ = 0.98). Pairwise comparisons showed significant increases in RPE from pre- to post-session in the BJ (p = 0.004, d = 5.98), CR (p = 0.003, d = 6.76), and PLA conditions (p = 0.003, d = 7.49) (Figure 3).

### Supplementation condition effects on ∆Pre-Post change

**FIG. 4:**
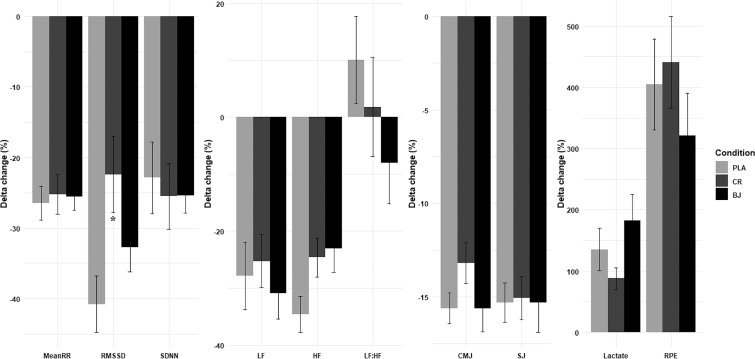
∆Pre-Post (%) for HRV indices, CMJ, SJ, lactate, and RPE. PLA: Placebo; CR: Creatine; BJ: Beetroot juice; *: compared to PLA.

Statistical analyses revealed non-significant supplementation condition ∆ pre–post for mean RR (F_(2, 30)_ = 0.07, p = 0.929, $\eta_{\text{p}}^{2}$ = 0.01), SDNN (test(2) = 1.27, p = 0.529, w = 0.06), LF (F_(2, 30)_ = 0.31, p = 0.738, $\eta_{\text{p}}^{2}$ = 0.02), HF (F_(2, 30)_ = 2.99, p = 0.065, $\eta_{\text{p}}^{2}$ = 0.16), LF:HF ratio (F_(2, 30)_ = 1.32, p = 0.284, $\eta_{\text{p}}^{2}$ = 0.08), CMJ (F_(2, 20)_ = 1.73, p = 0.194, $\eta_{\text{p}}^{2}$ = 0.1), SJ (F_(2, 30)_ = 0.01, p = 0.986, $\eta_{\text{p}}^{2}$ = 0), lactate (test(2) = 2.36, p = 0.307, w = 0.11), and RPE (test(2) = 3.82, p = 0.148, w = 0.174). However, there was a significant effect of supplementation condition in ∆ pre–post for RMSSD (F_(2, 30)_ = 4.45, p = 0.02, $\eta_{\text{p}}^{2}$ = 0.23), where the CR condition had a significantly lower ∆ *pre–post* compared to PLA (p = 0.017, d = 1.17) (Figure 4).

## DISCUSSION

This study aimed to investigate the effects of BJ and CR supplementation on maximal strength performance, HRV, and muscle oxygenation during incremental strength testing session consisted of two exercises (BP and BS). To our knowledge, this is the first study to directly compare the acute effects of BJ and CR on strength performance, HRV, and muscle oxygenation during resistance training, addressing a notable gap identified in the literature. The results partially confirmed our hypotheses. First, while CR and BJ both improved peak velocity compared to PLA, neither supplement demonstrated superiority in maximal strength performance over the other, as direct 1-RM comparisons were not tested. However, the observed peak velocity advantage in CR and BJ aligns with their proposed roles in anaerobic power and neuromuscular efficiency, respectively. Second, as hypothesized, BJ significantly enhanced SmO_2_ at all intensities compared to CR and PLA, supporting its vasodilatory and oxygen delivery benefits. Third, BJ also improved HRV metrics (e.g., RMSSD, SDNN, HF) both pre- and post-session relative to CR and PLA, confirming its cardiovascular efficiency advantages. Notably, CR showed no meaningful HRV effects, consistent with its anaerobic-focused mechanisms. Finally, the complementary roles of BJ and CR were partially supported: BJ uniquely improved oxygenation and HRV, while CR contributed to peak velocity. However, neither supplement mitigated fatigue-related declines in CMJ and SJ performance or lactate and RPE increases, suggesting their benefits are context-specific rather than broadly fatigue-resistant.

### Strength performance

These results align with previous findings indicating a natural decline in velocity and power output with increased resistance while suggesting that BJ and CR supplementation may provide benefits for maintaining movement speed under high loads. The declines in the number of repetitions performed, peak velocity, and peak power with increasing intensity from 60% to 80% of 1-RM across all supplementation conditions are consistent with the well-established loadvelocity and load-power relationships in resistance training [9, 11]. However, the significant enhancements in peak velocity at 60% and 70% of 1-RM under BJ and CR conditions, compared to PLA, suggest that these supplements may support velocity maintenance at submaximal intensities. This in the line with prior studies indicating that nitrate-rich supplementation, such as BJ, improves contractile efficiency and oxygen utilization, which preserves movement velocity with moderate loads [10, 16].

Furthermore, improvements in peak velocity and power failed to result in a significantly increased number of repetitions until failure for the BJ and CR conditions in the BP exercise. This contrasts with Williams, Martin [16], who reported higher endurance performance in resistance-trained males after BJ supplementation. The observed gains in peak velocity and power may reflect enhanced movement quality rather than true increases in maximal strength, which could account for the lack of improvement in repetition performance [69], which is consistent with CR’s phosphagen buffering that improves rapid force production without necessarily shifting the maximal load–velocity curve. Methodological differences between research could explain the difference. Contrary to Williams, Martin [16], our study focused primarily on the concentric phase of movement with a controlled isometric rest, which might have decreased feasible mechanical advantages and altered the velocity-load profile. Furthermore, the use of a Smith machine in our study likely increased measurement reliability while decreasing the natural stabilizing demands associated with free-weight exercises, which could have influenced overall performance outcomes [70].

The order in which the exercises are executed is a further factor influencing performance differences. In our protocol, the BP was performed before the BS, which may have influenced the observed results. While upper-body exercises such as the BP use smaller muscle groups and have a lower overall metabolic effort, doing them first may have helped individuals to sustain higher initial performance levels before neuromuscular fatigue set in in both lifts. BJ and CR supplementation improved peak velocity in the squat, suggesting their potential in mitigating fatigue-induced decreases in movement velocity. However, as participants switched to the BS, accumulated fatigue from the BP may have influenced lower-body performance. This is in line with previous findings that nitrate supplementation may preferentially improve lower-body performance due to its effects on oxygen availability and muscle perfusion [11, 71]. Furthermore, larger muscular groups involved in squatting are better prepared to endure lengthy contractions and recover from previous exertion, which could explain the observed trends in velocity and power production [72].

Despite the lack of significant improvements in endurance performance, the benefits of BJ and CR supplementation for velocity maintenance at moderate loads suggest potential advantages for powerbased training protocols.

Nitrate-derived nitric oxide enhances oxygen delivery and vasodilation, directing more blood to working muscles and boosting aerobic ATP resynthesis at sub-maximal loads [9, 10]. These changes help explain the faster velocity recorded at 60% and 70% of 1-RM, recruiting a large proportion of type II fibers. In contrast, maximal displacement remained unchanged, consistent with evidence that movement amplitude is governed mostly by neuromuscular coordination and individual biomechanics rather than dietary interventions [9]. On the other hand, CR operates through complementary, phosphagen-based pathways: it elevates intramuscular phosphocreatine stores, enabling faster ATP regeneration during the first seconds of high-intensity efforts; buffers cytosolic ADP and hydrogen ions, delaying fatigue; promotes cell swelling, which can up-regulate anabolic signalling; and may increase satellite-cell activity, supporting long-term strength and hypertrophy adaptations [19, 73]. Together, BJ and CR target distinct, but synergistic, energetic systems, potentially amplifying power output across a broader intensity spectrum.

Several strength performance outcomes in both BP and BS declined progressively as intensity increased across all supplementation conditions. While overall patterns were similar, no clear differences in repetitions performed were observed between the BJ, CR, and PLA groups at matched intensities. These findings contrast with previous studies that reported improved muscular endurance following BJ supplementation. Methodological discrepancies could be one probable cause; for instance, the use of a Smith machine in the present study may have constrained natural bar path and reduced stabilizer muscle involvement compared to free-weight protocols used elsewhere, which typically elicit higher neuromuscular activation and coordination demands [70]. Furthermore, the fixed exercise order may have contributed to fatigue accumulation, particularly affecting performance in exercises completed later in the session, an effect known to influence training adaptations and muscular endurance [74].

### Cardiovascular and Metabolic Responses

HR increased significantly with intensity across all conditions, with the BJ condition showing significantly lower HR compared to CR and PLA at higher intensities. The observed divergence in peak HR responses between the BJ, CR, and PLA conditions during incremental exercise underscores the unique cardiovascular and metabolic pathways modulated by these supplements. The significantly lower peak HR in the BJ condition at higher exercise intensities highlights the role of ${\text{NO}}_{3}^{-}$ in enhancing cardiovascular efficiency. Following ingestion, ${\text{NO}}_{3}^{-}$ is reduced to ${\text{NO}}_{2}^{-}$ by oral microbiota and further converted to NO under the hypoxic conditions of intense exercise [75]. NO acts as a potent vasodilator by activating soluble guanylate cyclase (sGC), which elevates cyclic guanosine monophosphate (cGMP) levels, leading to relaxation of vascular smooth muscle and reduced systemic vascular resistance [76]. NO-mediated vasodilation enhances perfusion to active muscles and the heart, increasing stroke volume via the Frank-Starling mechanism by boosting venous return and preload. This allows the heart to sustain cardiac output with fewer beats, explaining the reduced HR in the BJ [76]. Additionally, NO improves mitochondrial efficiency—likely by interacting with cytochrome c oxidase—lowering the oxygen cost of ATP production. As a result, less oxygen is needed for a given workload, reducing the metabolic stimulus for elevated HR [9, 75]. For instance, Larsen, Weitzberg [75] demonstrated that dietary nitrate reduces VO_2_ during submaximal cycling by 5–10%, paralleling the lower HR observed in BJ. This dual mechanism (vasodilation + mitochondrial efficiency) positions BJ as a potent ergogenic aid for endurance activities, where sustained cardiovascular efficiency is critical. On the other hand, CR supplementation, as we mentioned earlier, primarily augmenting intramuscular phosphocreatine stores to buffer ATP depletion during short-duration, high-intensity efforts via the creatine kinase reaction [19]. While CR enhances anaerobic power output in activities such as sprinting or resistance training, it lacks direct effects on vascular tone or systemic oxygen delivery. This explains why the CR condition’s HR profile remained the same as PLA during incremental exercise, a protocol dominated by aerobic metabolism. CR’s benefits are confined to rapid ATP regeneration in type II muscle fibers, offering no advantage in scenarios requiring sustained cardiovascular efficiency or oxygen utilization [19].

Additionally, SmO_2_ was consistently higher in the BJ condition compared to CR and PLA at all intensities, which may indicate enhanced muscle oxygenation and delayed onset of fatigue. The consistently higher SmO_2_ in the BJ condition across all exercise intensities further reinforces its role in optimizing oxygen dynamics [9]. Improved perfusion ensures a more homogeneous distribution of oxygenated blood, while NO’s modulation of the oxygen dissociation curve (via the Bohr effect) facilitates oxygen release from hemoglobin at lower tissue oxygen tensions [77]. This dual effect—enhanced delivery and extraction—delays the critical threshold at which oxygen supply fails to meet demand, preserving oxidative phosphorylation and attenuating lactate accumulation [78]. NIRS studies corroborate this, showing that BJ supplementation reduces the rate of muscle deoxygenation during incremental exercise, correlating with a 10–15% extension in time to exhaustion [5]. Moreover, CR’s lack of influence on SmO_2_ aligns with its anaerobic mechanism, which does not modulate oxygen kinetics or vascular function.

Furthermore, tHb showed no significant differences between conditions. This suggests that BJ’s benefits are not driven by increased total blood volume in the muscle but rather by improved distribution and extraction efficiency. NO’s vasodilatory effects likely optimize perfusion of previously underutilized capillaries, redirecting blood flow to metabolically active regions without altering overall tHb [9]. Similarly, CR’s absence of vascular effects explains its neutral tHb response.

### HRV responses

In examining the effects of BJ and CR supplementation on HRV parameters post-session, our findings suggest that BJ enhances autonomic recovery more effectively than both CR and PLA. Specifically, RMSSD and SDNN values were significantly higher in the BJ condition compared to PLA and CR, both before and after the session, though all conditions exhibited significant decreases in these metrics from pre- to post-session. These reductions in RMSSD and SDNN reflect the autonomic stress induced by increased exercise intensity, consistent with the suppression of vagal tone following high-intensity efforts [4]. BJ, however, showed the most favorable recovery profile, aligning with studies demonstrating improved cardiovascular recovery via nitric oxide-mediated vasodilation [5, 76].

Furthermore, LF and HF values decreased significantly post-session across all conditions. Notably, BJ had greater HF values than PLA, indicating that BJ supplementation may have helped mitigate the sympathetic dominance typically associated with high-intensity resistance exercise [79]. HF power, representing parasympathetic modulation, was significantly reduced across all conditions, with PLA showing the greatest decrease. This suggests the PLA condition resulted in the highest parasympathetic withdrawal, while BJ and CR maintained greater parasympathetic activity. The benefits of BJ align with the role of dietary nitrates in enhancing blood flow and reducing oxidative stress [76], whereas CR’s effects may relate to its ability to enhance phosphocreatine resynthesis and buffer energy demands during recovery [19].

LF, which represents both sympathetic and parasympathetic modulation, was strongly reduced across all conditions, with BJ showing the largest decrease, followed by CR. This could indicate statistically greater (Cohen’s d = 1.13–1.8) parasympathetic reboundand faster recovery following BJ and CR supplementation. BJ’s effects are consistent with nitric oxide’s role in improving autonomic balance [76], while CR’s benefits may stem from its capacity to reduce exercise-induced metabolic stress, thereby indirectly supporting autonomic stability [80]. Conversely, the PLA condition had a smaller reduction in LF, implying slower recovery compared to BJ and CR.

The LF:HF ratio, a marker of autonomic balance, slightly increased in the PLA condition, reflecting a stronger shift toward sympathetic activation, which suggests slower recovery [81]. However, the BJ and CR conditions showed a non-significant decrease in the LF:HF ratio, indicating a shift toward parasympathetic dominance and more favorable autonomic recovery. BJ supplementation, in particular, resulted in the greatest shift toward parasympathetic activity, providing the most favorable recovery profile, as seen in studies linking nitric oxide bioavailability to improved post-exercise autonomic regulation [76]. CR also supported recovery, albeit less effectively than BJ, likely due to its role in cellular hydration and phosphocreatine system support [19]. PLA resulted in the worst recovery, with the highest LF:HF ratio and greatest reduction in parasympathetic markers (RMSSD and HF), consistent with unmitigated exercise-induced stress [82].

These post-exercise HRV gains are most parsimoniously explained by a nitric-oxide–mediated up-regulation of arterial baroreflex sensitivity. Acute increases in endothelial and neuronal NO boost baroreceptor afferent firing and central baroreflex gain; this in turn expedites vagal re-engagement, so high-frequency HRV indices (e.g., RMSSD, HF power) rebound quickly even while sympathetic withdrawal is still minimal. Experimental NO blockade blunts both baroreflex gain and HF-HRV, whereas states with chronically low NO show parallel reductions in baroreflex sensitivity and vagal tone, underscoring the causal link between NO, baroreflex function and the speed of parasympathetic recovery [83].

### Lower limb strength

CMJ and SJ performance decreased significantly from pre- to postsession across all supplementation conditions, indicating increased neuromuscular fatigue following high-intensity resistance exercise. However, BJ and CR supplementation resulted in significantly better CMJ performance retention post-exercise compared to PLA, but not for SJ, likely because SJ relies solely on concentric force without a stretch–shortening cycle, making it less responsive to metabolic or elasticity-related ergogenic aids [84].

The significant reductions in CMJ and SJ performance observed across all supplementation conditions following high-intensity resistance exercise highlight the acute neuromuscular fatigue induced by such protocols. These declines are consistent with established mechanisms of fatigue, including metabolic by-product accumulation, transient muscle damage, and central nervous system adaptations [4, 85]. However, the attenuated decline in CMJ performance with BJ and CR compared to PLA suggests these interventions may support neuromuscular recovery or delay fatigue progression. The ergogenic effects of BJ are likely attributable to its high nitrate content, which is converted to NO in the body. NO as mentioned above enhances vasodilation, improves oxygen delivery, and increases mitochondrial efficiency, potentially mitigating fatigue by accelerating metabolite clearance and sustaining energy production during intense exercise [76, 78]. Prior studies report that nitrate supplementation preserves power output during repeated sprint and resistance exercise [5, 86], aligning with our findings that BJ may reduce fatigue-related declines in CMJ performance. This could reflect improved calcium handling or reduced oxidative stress in skeletal muscle [87].

Similarly, CR supplementation is well-established to enhance anaerobic capacity and recovery. By increasing phosphocreatine stores, CR buffers ADP accumulation and supports ATP regeneration during high-intensity efforts, which may delay peripheral fatigue and sustain neuromuscular performance [19]. The superior CMJ retention in the CR condition compared to PLA may derive from these mechanisms, as creatine’s role in stabilizing cellular energy turnover can preserve force production during fatiguing protocols. Furthermore, creatine-induced cellular hydration may help maintain muscle membrane stability and neuromuscular function under stress [19]. Nevertheless, the decrease of CMJ during the PLA condition underscores the absence of ergogenic support. Without BJ’s vasodilatory effects or CR’s energy-buffering properties, PLA participants likely experienced greater disruptions in excitation-contraction coupling and metabolite accumulation [88]. The lack of improvement in SJ performance across conditions may reflect its reliance on concentric force production without a stretch-shortening cycle, rendering it less responsive to interventions targeting metabolic or elastic recovery [85].

Overall, BJ and CR demonstrate potential as effective interventions to counteract neuromuscular fatigue during high-intensity training. The benefits of BJ are associated with nitric oxide-mediated improvements in vascular function and energy metabolism [76], whereas CR supports recovery by stabilizing cellular energy production [19]. Exploring the combined application of these supplements in future studies may uncover synergistic effects that further enhance recovery outcomes.

### Lactate and RPE

Lactate levels increased significantly post-session in all supplementation conditions, suggesting the metabolic demands of resistance training. Particularly, the CR condition showed significantly lower postexercise lactate levels than PLA. The reduction in lactate accumulation observed with CR can be linked to its role in replenishing phosphocreatine and functioning as an intracellular buffer [89]. Research indicates that CR supplementation helps minimize hydrogen ion production, a key factor in exercise-induced fatigue, thereby improving performance by reducing metabolic acidosis [90, 91]. Additionally, increasing intramuscular creatine reserves enhances the efficiency of anaerobic energy resynthesis, leading to greater reliance on glycolytic pathways for energy production. This process results in decreased anaerobic glycogen breakdown and modulates Ca^2+^-ATPase activity, facilitating faster muscle relaxation and improving the cross-bridge interaction between actin and myosin [92, 93]. Therefore, the present findings indicate that short-term CR supplementation plays a crucial role in minimizing lactate accumulation during resistance training, reinforcing its function as a buffering agent in active muscles.

BJ showed a moderate reduction in lactate accumulation, though slightly more than CR. Moreover, dietary nitrate from BJ has been found to improve metabolic efficiency, potentially lowering lactate levels by increasing mitochondrial respiration and oxygen use [9, 94]. RPE increased significantly post-session in all supplementation conditions, indicating a progressive accumulation of physiological and psychological fatigue. However, both the BJ and CR conditions resulted in significantly lower post-exercise RPE values than PLA, showing a potential reduction in RPE with supplementation. The effects of BJ on RPE remain contentious in the research. While a few studies found increased performance without significant adjustments in RPE [11, 95], other research found RPE decreases with BJ. Our findings are consistent with Mosher, Sparks [11], who discovered no significant effect of BJ on RPE during resistance exercise, despite improvements in performance. However, the discrepancy between improved SmO_2_ and unchanged RPE mirrors reports that local oxygenation does not always translate to lower global effort perception, which is modulated by central and motivational factors [16].

One speculative explanation is increased cerebral perfusion; however, we did not measure cerebral blood flow and thus refrain from firm conclusions [78]. Furthermore, preserving RPE despite enhanced performance could be attributable to a decrease in central motor command due to preserved contractile function [96]. Given that RPE is a centrally mediated feedback system where a copy of the central motor command is sent to sensory areas for conscious awareness [97], BJ supplementation might maintain neuromuscular function while maintaining comparable perceived effort levels. In contrast, CR supplementation showed lower post-exercise lactate levels and RPE, indicating that it may be more effective in reducing neuromuscular fatigue and metabolic stress. This finding is in line with previous research showing that CR improves ATP resynthesis, delays fatigue, and decreases perceived exercise difficulty [24, 31]. Although CR supplementation reduced blood lactate levels, it did not alter RPE. This may be attributed to the fact that even a short creatine loading protocol increases intramuscular free creatine and phosphocreatine stores. Through the creatine kinase reaction (H^+^ + Cr + ADP → PCr + ATP), protons are consumed, buffering intracellular pH and slowing glycolytic flux, thereby attenuating the exercise-induced rise in lactate [98, 99]. However, RPE reflects the brain’s integration of various central and peripheral cues, not just peripheral acidosis, so the central perception of effort can remain unchanged despite reduced lactate accumulation [100, 101].

Thus, while both CR and BJ have ergogenic benefits, CR appears to be more effective in buffering metabolic acidosis and reducing perceived exertion, whereas BJ could enhance performance tolerance without significantly affecting RPE.

### Practical Applications

In practical terms, athletes and coaches can consider incorporating nitrate-rich BJ and creatine into their training routines to potentially enhance performance and recovery. For example, consuming beetroot juice about 2 hours before a workout may improve muscle endurance by boosting oxygen delivery, and maintaining a regular creatine supplementation schedule can increase strength and power over time. It’s also important to balance machine-based exercises with free-weight training: while the Smith machine can help with form and safety, adding free-weight lifts (like traditional barbell squats and presses) will engage stabilizer muscles and better translate to functional, real-world strength. Finally, coaches should be mindful of exercise order and fatigue management in workouts, and ensure athletes stay hydrated and follow consistent pre-workout routines to maximize the benefits of any supplementation.

### Limitations

The study’s design introduced several constraints that impact realworld applicability. Using a Smith machine and a fixed exercise order (bench press always preceding back squat) prioritized reliability over ecological validity by limiting stabilizer muscle engagement and potentially causing fatigue carryover into the lower-body exercise. Additionally, the incremental loading protocol (60–80% 1-RM) may have induced accumulated fatigue, and focusing only on acute supplementation effects might not reflect the chronic adaptations possible with longer-term beetroot juice or creatine use. In terms of measurements, the protocol did not confirm supplement bioavailability: plasma ${\text{NO}}_{2}^{-}$ and intramuscular creatine levels were not measured, leaving uptake effects unverified. Despite external validation of the beetroot juice’s nitrate content, the creatine composition was not independently verified. The study also relied on self-reported dietary compliance without objective checks, and the lack of detailed reporting on adherence to dietary restrictions, oral hygiene, hydration, physical activity, and sleep introduces potential recall bias regarding true compliance. Furthermore, our physiological measurements had a limited scope: NIRS monitoring was restricted to superficial muscle oxygenation (omitting deeper tissue dynamics), subjective RPE lacked objective correlates (e.g., electromyography), and we did not analyze combined BJ andCR effects or account for real-world recovery factors (such as hydration status or environmental stress), all of which further constrain the findings’ applicability. Finally, the sample size was small (only 11 resistance-trained male participants), which severely limits the statistical power and generalizability of the results to other populations (e.g., untrained individuals, women, or athletes in different disciplines). Future studies should address these limitations by integrating biomarker validation (measuring plasma nitrite and creatine levels), incorporating chronic supplementation protocols, employing free-weight exercise designs for greater ecological validity, recruiting larger and more heterogeneous cohorts, and including deeper tissue monitoring alongside real-world recovery variables in their analyses.

## CONCLUSIONS

This study demonstrates that BJ and CR supplementation differentially enhance recovery and performance during high-intensity resistance exercise. BJ improved cardiovascular efficiency and autonomic recovery via nitric oxide-mediated vasodilation and mitochondrial efficiency, evidenced by lower heart rates, higher SmO_2_, and superior post-exercise HRV compared to CR and PLA. CR supported anaerobic performance by buffering metabolic stress, reducing lactate accumulation, and maintaining peak velocity at moderate intensities. Both supplements attenuated declines in CMJ performance post-exercise, though SJ remained unaffected, likely due to neuromuscular demand differences. These findings suggest BJ is advantageous for enduranceoriented protocols, while CR benefits power-focused efforts, with future research needed to explore their combined use and chronic effects.

## Data Availability

The data supporting this study are available upon request from the corresponding author.
