# Correction: Acute effects of beetroot juice vs. creatine supplementation on maximal strength, autonomic regulation, and muscle oxygenation during incremental resistance exercise

**DOI:** 10.5114/biolsport.2026.156734

**Published:** 2025-12-03

**Authors:** Atef Salem, Achraf Ammar, Mohamed Kerkeni, Mohamed Ali Boujelbane, Ayse Merve Uyar, Leonard Moritz Köbel, Saranya Selvaraj, Reza Zare, Katie M. Heinrich, Haitham Jahrami, Slim Tounsi, Giuseppe Grosso, Piotr Zmijewski, Wolfgang I. Schöllhorn, Khaled Trabelsi, Hamdi Chtourou

**Affiliations:** 1 Department of Training and Movement Science, Institute of Sport Science, Johannes Gutenberg-University Mainz, 55122 Mainz, Germany; 2 High Institute of Sport and Physical Education of Sfax, University of Sfax, Sfax 3000, Tunisia; 3 Research Laboratory, Molecular Bases of Human Pathology, LR19ES13, Faculty of Medicine of Sfax, University of Sfax, Sfax 3000, Tunisia; 4 Research Laboratory: Education, Motricity, Sport and Health, EM2S, LR19JS01, High Institute of Sport and Physical Education of Sfax, University of Sfax, Sfax 3000, Tunisia; 5 Department of Chemistry, Faculty of Applied Sciences, University of Sri Jayewardenepura, Gangodawila, Nugegoda, Sri Lanka; 6 SRH Campus Hamburg, SRH University of Applied Sciences Heidelberg, 20095 Hamburg, Germany; 7 Department of Kinesiology, Kansas State University, Manhattan, KS 66506, USA; 8 Department of Research and Evaluation, The Phoenix, Denver, CO 80205, USA; 9 Government Hospitals, Manama, Bahrain; 10 Department of Psychiatry, College of Medicine and Medical Sciences, Arabian Gulf University, Manama, Bahrain; 11 Laboratory of Biopesticides (LBPES), Center of Biotechnology of Sfax, University of Sfax, Sfax, Tunisia; 12 Department of Biomedical and Biotechnological Sciences, University of Catania, Catania, Italy; 13 Jozef Pilsudski University of Physical Education in Warsaw, 00-809 Warsaw, Poland; 14 Department of Movement Sciences and Sports Training, School of Sport Science, The University of Jordan, Amman, Jordan; 15 Research Unit, Physical Activity, Sport, and Health, UR18JS01, National Observatory of Sport, Tunis 1003, Tunisia

**Keywords:** Nitrate, Ergogenic effect, Dietary Supplements, EIMD, Strength, Recovery

## Erratum:

In the published article, an error was identified in Table 3. Table 3 presented the results of strength-test performances in the bench press and back squat across incremental intensities among novice resistance-trained subjects, including differences by supplementation and comparisons between conditions and intensities. However, the table mistakenly contained incorrect data for the number of maximum repetitions in the back squat across the three supplementation conditions and intensities.

The corrected Table 3 is provided below.

**TABLE 3:** ANOVA/LD.F2 Results for Strength Test Performances in Bench Press and Back Squat at Incremental Intensities, with Differences by Supplementation and Comparisons Between Conditions and Intensities.

	Supplementation Condition	BP			ANOVA/LD.F2	BS			ANOVA/LD.F2
		
		60%	70%	80%		60%	70%	80%	
**Maximum Repetitions**	**PLA**	12.73 ± 2.94	7.27 ± 1.9 *	3.09 ± 1.45 *¤	**SC:** F_(1.77, 17.70)_ = 7.04, p = 0.007, η^2^_p_ = 0.413	13.73 ± 6.97	10.00 ± 5.00	6.00 ± 3.69 *	**SC:** F_(1.44, 14.28)_ = 5.72, p = 0.022, η^2^_p_ = 0.36

	**CR**	15.09 ± 4.13	10.18 ± 4.35 *	4.18 ± 2.18 *¤	**I:** F_(1.85, 18.55)_ = 198.29, p < 0.001, η^2^_p_ = 0.952	20.09 ± 8.68	11.45 ± 4.63 *	8.09 ± 4.44 *	**I:** F_(1.61, 16.06)_ = 48.25, p < 0.001, η^2^_p_ = 0.83

	**BJ**	15.45 ± 4.41	9.18 ± 3.74 *	5 ± 3.03 *¤	**SC × I:** F_(2.87, 28.66)_ = 0.97, p = 0.418, η^2^_p_ = 0.088	19.64 ± 9.28	13.09 ± 7.44	9.00 ± 4.63 *	**SC × I:** F_(2.71, 27.09)_ = 2.23, p = 0.113, η^2^_p_ = 0.18

**Peak Velocity (m · s−1)**	**PLA**	0.56 ± 0.08	0.44 ± 0.07 *	0.32 ± 0.06 *¤	**SC:** F_(1.64, ∞)_ = 40.75, p < 0.001, η^2^_p_ = 0.80	0.55 ± 0.1	0.49 ± 0.06	0.35 ± 0.06 *¤	**SC:** F_(1.59, ∞)_ = 37.18, p < 0.001, η^2^_p_ = 0.79

	**CR**	0.66 ± 0.07	0.54 ± 0.07 *	0.48 ± 0.06 *^a^	**I:** F_(1.87, ∞)_ = 72.48, p < 0.001, η^2^_p_ = 0.88	0.72 ± 0.09 a	0.58 ± 0.07 *	0.5 ± 0.06 *¤^a^	**I:** F_(1.59, ∞)_ = 119.26, p < 0.001, η^2^_p_ = 0.92

	**BJ**	0.72 ± 0.07 a	0.56 ± 0.08 *^a^	0.5 ± 0.08 *^a^	**SC × I:** F_(3.09, ∞)_ = 0.10, p = 0.961, η^2^_p_ = 0.01	0.64 ± 0.11	0.59 ± 0.09 a	0.46 ± 0.09 *¤^a^	**SC × I:** F_(2.7, ∞)_ = 1.94, p = 0.127, η^2^_p_ = 0.16

**Peak Power (W)**	**PLA**	508.24 ± 61.07	577.91 ± 57.73 *	767.99 ± 66.19 *¤	**SC:** F_(1.54, 15.38)_ = 0.90, p = 0.401, η^2^_p_ = 0.08	878.67 ± 49.87	1078.35 ± 60.52 *	1358.47 ± 81.45 *¤	**SC:** F_(1.83, ∞)_ = 1.58, p = 0.208, η^2^_p_ = 0.14

	**CR**	510.28 ± 57.17	617.88 ± 53.05 *	787.23 ± 62.66 *¤	**I:** F_(1.97, 19.68)_ = 251.52, p < 0.001, η^2^_p_ = 0.96	926.34 ± 38.17	1095.93 ± 59.45 *	1331.77 ± 97.25 *¤	**I:** F_(1.91, ∞)_ = 536.64, p < 0.001, η^2^_p_ = 0.98

	**BJ**	503.09 ± 69.57	594.65 ± 58.3 *	787.65 ± 49.57 *¤	**SC × I:** F_(2.90, 28.98)_ = 0.31, p = 0.810, η^2^_p_ = 0.03	849.11 ± 46.38 b	1092.39 ± 33.52 *	1347.83 ± 69.78 *¤	**SC × I:** F_(3.08, ∞)_ = 2.09, p = 0.097, η^2^_p_ = 0.17

**Maximum Displacement (cm)**	**PLA**	46.73 ± 2.57	39.82 ± 3.92 *	37.18 ± 1.66 *	**SC:** F_(1.78, ∞)_ = 0.42, p = 0.634, η^2^_p_ = 0.04	54.36 ± 2.11	49.73 ± 3.82 *	47.55 ± 2.11 *	**SC:** F_(1.90, ∞)_ = 2.22, p = 0.111, η^2^_p_ = 0.18

	**CR**	45.27 ± 4	41.18 ± 3.63	38.45 ± 1.44 *	**I:** F_(1.26, ∞)_ = 48.97, p < 0.001, η^2^_p_ = 0.83	54.64 ± 2.62	50.27 ± 3.1 *	47.27 ± 1.56 *¤	**I:** F_(1.63, ∞)_ = 112.31, p < 0.001, η^2^_p_ = 0.92

	**BJ**	45.73 ± 2.1	40.18 ± 3.57 *	37.73 ± 1.85 *	**SC × I:** F_(2.76, ∞)_ = 1.05, p = 0.364, η^2^_p_ = 0.10	56.09 ± 2.91	51.64 ± 2.98 *	47.73 ± 1.49 *¤	**SC × I:** F_(2.59, ∞)_ = 0.21, p = 0.866, η^2^_p_ = 0.02

**BP**: Bench press; **BS**: Back squat; **PLA**: Placebo; **CR**: Creatine; **BJ**: Beetroot juice; **SC:** main effect of supplementation condition; **I:** Main effect of intensity; **SC × I:** interaction between supplementation condition and intensity;

* : compared to 60%;

¤ : compared to 70%;

a : compared to PLA;

b : compared to CR.

